# Interim Safety and Biomarker Data From upliFT‐D Trial of PBFT02 in FTD with GRN Mutations

**DOI:** 10.1002/alz70859_103638

**Published:** 2025-12-25

**Authors:** Tiffini Voss, Paulette Triglia, Yan G Ni, Sue Browne, William Chou, Simon Ducharme, David J. Irwin, Isabel Santana, Paul E Schulz, Leonel Tadao Takada, Carmela Tartaglia, Leonardo Cruz de Souza

**Affiliations:** ^1^ PassageBio, Philadelphia, PA USA; ^2^ Passage Bio, Philadelphia, PA USA; ^3^ Montreal Neurological Institute, McGill University, Montreal, QC Canada; ^4^ Perelman School of Medicine, University of Pennsylvania, Philadelphia, PA USA; ^5^ Neurology Department, Centro Hospitalar e Universitário de Coimbra, Coimbra, ‐ Portugal; ^6^ John P. and Kathrine G. McGovern Medical School at UTHealth, Houston, TX USA; ^7^ Hospital das Clínicas, University of Sao Paulo Medical School, São Paulo, São Paulo Brazil; ^8^ University of Toronto, Toronto, ON Canada; ^9^ Universidade Federal de Minas Gerais, Belo Horizonte Brazil

## Abstract

**Background:**

Approximately 25‐30% of frontotemporal dementia (FTD) is caused by autosomal dominant mutations, usually in one of three genes, *C9orf72*, GRN, or MAPT. There are currently no disease‐modifying treatments approved for FTD, including FTD‐GRN. PBFT02 is being developed as a disease‐modifying therapy for patients with FTD‐GRN and FTD‐*C9orf72*.

**Methods:**

PBFT02 is an AAV1 vector that delivers a copy of the human GRN gene directly to the CSF via a single intra‐cisterna magna (ICM) administration. PBFT02 is being studied in a first‐in‐human clinical trial, upliFT‐D (NCT04747431 [ClinicalTrials.gov]), which will sequentially enroll three FTD‐GRN cohorts and two FTD‐*C9orf72* cohorts. The primary objective is safety and tolerability; secondary objectives include biomarkers of target engagement (e.g., progranulin; PGRN), biological activity, and disease progression. The 2‐year trial will be followed by a 3‐year extension for safety and durability of effect.

**Results:**

As of December 2024, 7 participants received PBFT02 Dose 1 (3.3 E+10 genome copies/g brain weight, or 4.50 E+13 total genome copies), with an immunosuppressive regimen of oral prednisone 60mg for 30 days (N=1) or 1g IV methylprednisolone on study days 1‐3, followed by 60mg oral prednisone on days 4‐60 (N=6). PBFT02 increased CSF PGRN in all participants, from below 3 ng/mL at baseline to 13 – 27 ng/mL at six months (n=4) and 22 – 34 ng/mL at 12 months (n=2). Responses were stable and durable at 12 (N=2) and 18 months (N=1). In contrast, plasma PGRN remained below the mean healthy reference values in all participants to date. Plasma NfL levels were stable at 12 months (N=2) and demonstrated a reduced rate of change when compared to published natural history data. Two of 7 participants experienced a total of 3 SAEs, with 1 episode of hepatotoxicity and 2 episodes of asymptomatic venous sinus thrombosis. All remaining patients in cohort 2 will be treated with Dose 2, a 50% lower dose than Dose 1.

Updated data will be included at the time of the presentation.

**Conclusions:**

Interim safety and biomarker data from the upliFT‐D trial provides early evidence that PBFT02 has potential as a one‐time therapy for FTD‐GRN, thus supporting further clinical development.

